# The rise of online gambling addiction: A mental health challenge

**DOI:** 10.1192/j.eurpsy.2025.308

**Published:** 2025-08-26

**Authors:** R. M. Cabral, C. Cunha, P. Pires, I. Santos, F. Cunha, A. Oliveira, N. Gil, F. Coias

**Affiliations:** 1 Psychiatry, ULS-Viseu Dão Lafões, Viseu; 2 Family Medicine, ULS-Alentejo Central, Evora, Portugal

## Abstract

**Introduction:**

Gambling disorder is a rising concern among young adults, highlighting the need for effective screening to offer appropriate support and intervention.

**Objectives:**

This study aims to characterize gambling disorder among young adults (ages 18-25) in Portugal.

**Methods:**

A quantitative cross-sectional study was conducted using a self-administered online questionnaire completed by young adults.

**Results:**

This study included a population of 554 participants, 166 of whom were gamblers. Among the gamblers, the prevalence was as follows: 63% did not show signs of pathological gambling, while the remaining participants exhibited gambling addiction at varying levels: 25% mild, 9% moderate, and 3% severe. The typical profile of a gambler was identified as a male university student with an average age of 23.5 years, of a middle economic status and residing in an urban area. The preferred types of gambling were sports betting and online casino games. Most online gamblers had previously engaged in offline gambling at the age of M=19.25. The primary attractions of online gambling for these individuals were accessibility, the variety of games, and the potential for economic gains. The main encouragements to gamble online were friend´s influence and online advertising. No significant differences were observed in depression (PHQ-9) and anxiety scores (GAD-7) between gamblers and non-gamblers (p > 0.05). However, among gamblers, a strong positive correlation was found between higher levels of addiction (assessed by DSM-V gambling disorder criteria) and both depression and anxiety scores (r = 0.732, r = 0.681; p < 0.01). Furthermore, severe gambling cases were associated with a higher prevalence of prior formal diagnoses of psychiatric disorders, such as ADHD, anxiety, and depression, although this association was not statistically significant (p > 0.05). All gamblers showed a higher prevalence of substance abuse (p < 0.01). However, this trend did not extend to alcohol consumption (p > 0.05). The Jacobs Dissociative Experiences Scale was used to assess the presence of dissociative symptoms in relation to the severity of gambling addiction, revealing a strong positive correlation (r = 0.721; p < 0.01). Gamblers reported negative impacts on their family and romantical relationships. In contrast, they did not perceive their gambling behavior as having an adverse effect on their friendships or work performance. The majority (71.4%) of high-severity gamblers did not seek professional help and were not receiving any psychopharmacological therapy.

**Conclusions:**

This study features the critical need for effective screening and intervention strategies for gambling disorder among young adults, given its correlation with mental health disorders and substance abuse. Addressing these challenges is essential for enhancing support and improving outcomes for individuals with gambling disorder.

**Disclosure of Interest:**

None Declared

